# Multiple Retained Needles in the Chest and Abdomen Due to Self-Injury: A Case Report

**DOI:** 10.7759/cureus.76590

**Published:** 2024-12-29

**Authors:** Yasuaki Maeda, Kenichi Nitta, Hiroshi Kamijo, Hiroshi Takayama, Hiroshi Imamura

**Affiliations:** 1 Department of Emergency and Critical Care Medicine, Shinshu University Hospital, Matsumoto, JPN

**Keywords:** cardiac effusion, critical care, psychiatry, retained needles, schizophrenia, self-injury

## Abstract

Retained needles are rarely observed in multiple locations. Furthermore, although case reports on retained needles have been published, there are no standardized guidelines for managing retained needles. A 42-year-old man with schizophrenia was referred to our hospital for intensive care because of a pericardial effusion and 12 needles from needle pricks being retained in both his chest and abdomen. First, he underwent emergency pericardial drainage. Thereafter, he underwent surgeries to remove a needle from the pericardium on day 7, abdominal needles on day 14, and intrapulmonary needles on day 30. He was transferred to a psychiatric ward on day 36 after it was confirmed that no needles remained in the body. We report a case of multiple needles being retained in multiple locations due to self-injury caused by schizophrenia. Future guidelines are needed to help clinicians manage patients with retained needles.

## Introduction

Needles are a specific type of foreign body (FB) that are used in the medical field as well as in the general population. Prompt localization and removal are recommended due to the potential risk of injury associated with the sharp ends of needles [1]. Meanwhile, controversy exists about optimal treatment in asymptomatic patients in whom the risk of embolization or infection is low and a surgical procedure can be more dangerous than conservative treatment [2]. Furthermore, if multiple foreign bodies are in various locations, the order in which they are removed and the least invasive methods will differ. Although some case reports of retained needles have been reported, to our knowledge, there are no standardized guidelines for the management of retained needles [3].

Herein, we present a case of self-inflicted retained needles in multiple locations, requiring three surgeries for complete removal.

## Case presentation

**Figure 1:**
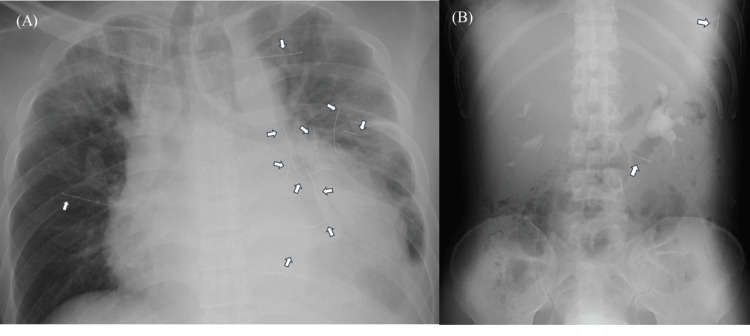
Chest and abdominal radiographs at admission. (A), (B) The chest and abdominal radiographies upon admission revealed cardiomegaly and several foreign bodies (white arrows),  consistent with retained needles in the patient’s chest and abdomen.

A 42-year-old male patient with a history of schizophrenia was found lying immobile on the street with difficulty moving. He was transported to a nearby community hospital, where computed tomography (CT) revealed multiple retained needles and a large pericardial effusion. His vitals were stable and he was given an intravenous infusion because emergency treatment was not possible there. He was transferred to our hospital for further treatment, given his history of self-injury and prior surgeries for retained intrathoracic needles. Upon arrival, his Glasgow Coma Scale score (level of consciousness) was 14 (E4V4M6). His vital signs included a heart rate of 104 beats per minute (bpm), a blood pressure of 163/100 mm Hg, respiratory rate of 20 breaths per minute (breaths/min), oxygen saturation of 94% on room air, and temperature of 37.6 degree Celsius. On physical examination, coarse crackles were auscultated in the left lower chest with numerous, non-suppurative, pigmented wounds noted to the anterior chest and no subcutaneous emphysema. No jugular venous distention was noted. Diagnostic studies including chest and abdominal X-rays were performed revealing cardiomegaly, several retained intrathoracic and intraabdominal foreign bodies and a slightly deviated trachea to the right (Figure 1A,B). An electrocardiogram (ECG) was performed and notable for low voltage, which was concerning for pericardial effusion. Laboratory test findings included anemia, mildly elevated aspartate transferase/alanine transaminase (AST/ALT), hyponatremia, elevated brain natriuretic hormone (BNP), and C-reactive protein levels (CRP). However, procalcitonin was negative (Table 1).

**Table 1:** Laboratory results at admission

Laboratory exam variables	Patient value	Reference range
White blood cells (WBC), 10*3/uL	4560	3300-8600
Hemoglobin, g/dL	8.5	13.7-16.8
Hematocrit, %	22.9	40.7-50.1
Platelet, 10*3/uL	34.6	15.8-34.8
Creatinine, mg/dL	0.48	0.65-1.07
Urea nitrogen, mg/dL	5.2	8.0-20.0
Total bilirubin, mg/dL	0.51	0.40-1.50
Aspartate transferase (AST), IU/L	70	13-30
Alanine transaminase (ALT), IU/L	86	10-42
Lactate dehydrogenase, IU/L	362	124-222
Alkaline phosphatase, IU/L	160	38-113
Creatinine kinase, IU/L	124	59-248
Sodium, mmol/L	121	138-145
Chlorine, mmol/L	90	101-108
C-reactive protein, mg/dL	15.67	0.00-0.14
Troponin-T, mg/dL	0.005	0.000-0.090
Prothrombin time, INR	1.25	0.85-1.15
Activated partial thromboplastin time, sec	31.4	23.0-38.0
D-dimer, μg/mL	10.7	0.0-1.0
Brain natriuretic hormone, pg/ml	82.4	0.0-20.0
Procalcitonin, ng/mL	0.02	0.00-0.49

**Figure 2:**
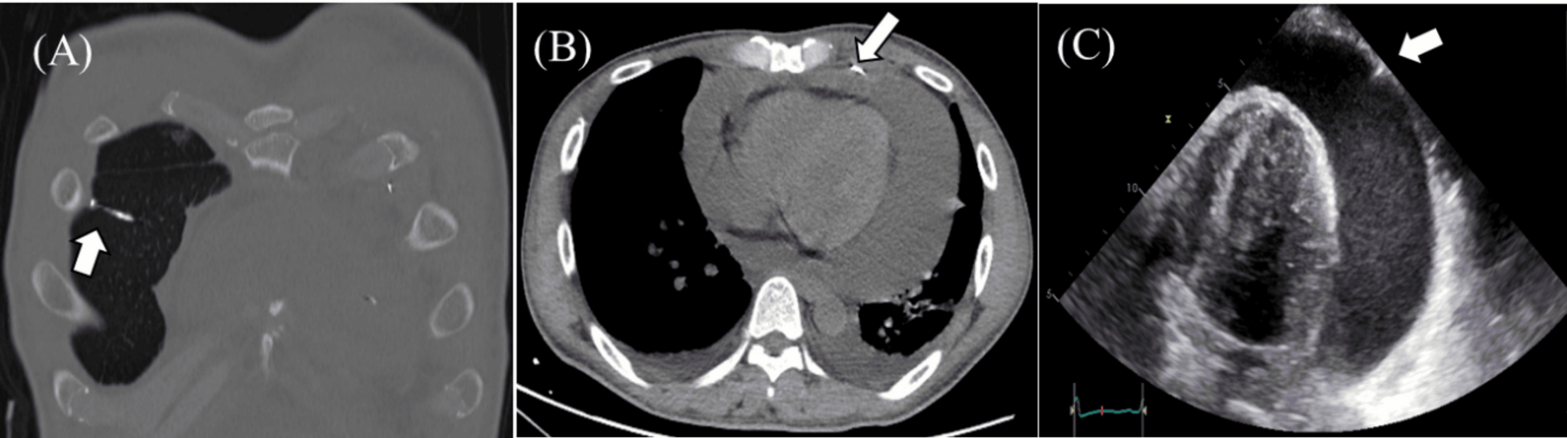
Chest computed tomography (CT) and cardiac ultrasound images at admission. (A) CT revealed a needle (white arrow) in the right lung parenchyma. (B) CT revealed a needle (white arrow) that had penetrated the pericardium. (C) Transthoracic echocardiography revealed a pericardial effusion of more than 500 mL and a hyperechoic sharp object (white arrow) in the parietal pericardium.

The CT of the chest without contrast performed at the previous hospital noted 12 retained needles - one in the right lung (Figure 2A), two in the left lung, seven in the anterior chest wall, and two in the abdomen. One of the chest wall needles seemed to penetrate the pericardium (Figure 2B). Infiltrative shadows were observed in both lungs, along with a pericardial effusion of more than 500 mL. Transthoracic echocardiography revealed a pericardial effusion of more than 500 mL and a hyperechoic pointed object in the parietal pericardium (Figure 2C). According to the patient’s interview, he had a history of excessive water intake. Water intoxication was suspected as the cause for hyponatremia. Water restriction was implemented. Importantly, he had engaged in self-injury and inserted the needles during the month before admission.

Without signs of cardiac tamponade, we determined the pericardial effusion was a chronic accumulation. Pericardial drainage was performed and a drain was placed. An amount of 600 mL of bloody effusion was drained from the pericardium initially. Based on elevated CRP and infiltrative shadows on chest x-ray, pneumonia was diagnosed. Intravenous administration of sulbactam/ampicillin (9 g/day) for pneumonia was initiated on the day of hospitalization. Reddish brown drainage of 60-300 mL/day continued thereafter. On day 7, a CT-guided fluoroscopic thoracotomy was performed. One needle was removed from the surrounding tissue without breaking the foreign body (FB). Postoperatively, the volume from the pericardial drain decreased, and the drain was removed on day 9.

Laparoscopic abdominal surgery was performed on day 14. A radiograph revealed two needles in the abdominal cavity (Figure 1B). Under fluoroscopic guidance, a needle was found within a nodule surrounded by the omentum. It was removed by excising the section of the omentum but the FB was eroded and broken. The other needle was located between the intercostal muscles near the diaphragm, rather than in the abdominal cavity, under fluoroscopic guidance and was removed percutaneously.

On day 30, fluoroscopic-guided thoracoscopy was performed. Small incisions in the lung parenchyma were performed, with each needle removed. A total of nine needles had been inserted into the chest cavity, with one in the right middle lobe and two in the left lingual area. Of the remaining six needles, five were subcutaneous, and one was embedded in scar tissue within the intercostal muscle, all of which were successfully removed. Finally, fluoroscopy confirmed the absence of any remaining needles within the body. Bilateral chest drains were inserted, and the surgery was completed. On day 31, we confirmed no pneumothorax based on radiographs or air leaks in either lung. The right and left chest drains were removed on days 33 and 34, respectively. On day 36, the patient was transferred to a psychiatric ward and, on day 42, to a family psychiatric hospital for involuntary psychiatric hospitalization.

## Discussion

We report a case of multiple needles being retained in a patient’s trunk due to self-injury caused by schizophrenia.

Needles, used both in medical and general settings, can become FBs. Three retention patterns exist, namely ingestion, iatrogenic retention, and penetration. Although uncommon, needle penetration is a known cause of retention, often accidental, particularly among tailoring workers. Intentional penetration is a rare incidence; however, it occurs more commonly in children and adults with mental disorders. Deep penetration in the abdomen or thorax, although not well-documented, is a potential risk [1]. Less frequently, extravascular FBs can migrate into the venous circulation and to the heart [4,5]. Approximately 30% of cases involve multiple needles [6].

There are no standardized guidelines for managing retained FBs [3,7]. However, it is generally agreed that symptomatic and asymptomatic FBs pose future risks and should be removed [8,9]. Retained needle-induced pericardial effusions require prompt identification and treatment to prevent hemodynamical complications, pericardial tamponade, and mortality risks. Emergency thoracotomy is required when pericardial tamponade is identified. For those without signs of cardiac tamponade, early surgical removal of the FB is recommended to prevent secondary symptoms [9]. At first, we decided to remove the retained needles, which was the cause of the pericardial effusion. Various surgical approaches can be used depending on the clinical scenario, including median sternotomies, and thoracotomies with and without cardiopulmonary bypass [2,10,11]. Given that there is no standard surgical incision for retrieving an FB from the heart, it would be better to begin with a smaller incision to assess the feasibility of completing the procedure [12]. Removal of FBs from the superior vena cava and right atrium without thoracotomy may also be performed under fluoroscopic guidance with an arterio-venous sheath placement for extracorporeal membrane oxygenation in some cases [2]. In our case, we successfully removed the retained needle under fluoroscopic guidance through a small left thoracotomy. Similarly, the intra-abdominal and intrathoracic needles could be removed laparoscopically and thoracoscopically, respectively.

There have been cases where FB has remained in situ for years without complications [2,8,13,14]. Moreover, debate still exists regarding the removal of FBs when the diagnosis is delayed [2]. Less frequently, extravascular FBs migrate into the venous circulation and to the heart [4,5]. Long-term FBs in the abdominal cavity (8 days to 10 years) may form granulation tissue or abscesses and may cause organ injury [15]. Therefore, we decided to remove all remaining needles in the thoracic and abdominal cavities.

Self-harm is indeed a significant global problem. The prevalence of self-harm, particularly among adolescents and young adults, is alarmingly high worldwide [16]. Self-injury by needle accounted for 3.6% of all self-injury. Self-inflicted intra-cardiac needle injuries occur mainly in young and middle-aged adults with psychiatric disorders, commonly depression, schizophrenia, and substance use disorders. Recurrent incidences of self-harm are rare [6]. In our case, the patient had a history of self-injury and surgery for intrathoracic needles. Therefore, continuing treatment for schizophrenia is crucial for his future well-being.

## Conclusions

We report a case of successful removal of multiple retained needles in a patient with schizophrenia through minimally invasive surgical approaches including thoracoscopy and laparoscopy under CT and fluoroscopic guidance. The patient presented with pericardial effusion and multiple needles in the chest and abdomen, which were completely removed through staged operations under appropriate imaging guidance. While there are no standardized guidelines for managing retained needles, our case demonstrates that a systematic surgical approach can achieve complete removal even in complex cases with multiple foreign bodies. Long-term psychiatric care remains crucial for preventing recurrent self-injury in such cases.
